# Navigating Intimate Physical Examination: A Qualitative Study of OBGYN Residents’ Experiences of Supervising Medical Students

**DOI:** 10.5334/pme.2306

**Published:** 2026-11-03

**Authors:** Chonkar Sonali Prashant, Jean-Jasmin Mi-Li Lee, Manisha Mathur, Ni Ni Soe, Tan Wei Mun Amanda, Predeebha Kannan, Janneke Frambach

**Affiliations:** 1 Department of Obstetrics and Gynaecology, KK Women’s and Children’s Hospital, Singapore; 2 Family Medicine Service, KK Women’s and Children’s Hospital, Singapore; 3 National Healthcare Group Polyclinics, Singapore; 4 School of Health Professions Education (SHE), Maastricht University, The Netherlands

## Abstract

**Introduction:**

Obstetrics and Gynaecology (OBGYN) residents are often responsible for medical students fulfilling learning objectives during clerkship, including performing pelvic examinations. Limited research exists on OBGYN residents’ experiences in supervising students performing these intimate examinations. This study explores how OBGYN residents navigate this supervisory role through the lens of the Cognitive Apprenticeship Model (CAM) by investigating their experiences supervising students performing these examinations on patients.

**Methods:**

This qualitative study, conducted at a single tertiary hospital in Singapore, purposively sampled ten OBGYN residents, reaching thematic sufficiency. Data were collected via one-on-one semi-structured interviews, informed by CAM and analyzed thematically using the model as a sensitizing framework. Reflexivity meetings were held for transparency of findings and interpretations.

**Results:**

Two overarching themes captured residents’ supervisory experiences: (1) challenges and support related to nuances of pelvic examinations, and (2) limited opportunities for students to perform them. Residents helped students, who particularly struggled with speculum examinations, by providing stepwise guidance using CAM components. Students’ exposure to bimanual examinations was restricted, as residents took over to reduce patient discomfort. Supervision was further constrained by consent issues, student factors, and systemic barriers. Residents employed CAM strategies to overcome these challenges.

**Discussion:**

Residents’ supervision of pelvic examinations were influenced by patient, student, and system factors. Despite this, residents used CAM strategies when opportunities arose, providing stepwise coaching, role modelling, reflection, and exploring ways to improve supervision. These findings offer valuable insights for healthcare educators designing Resident-as-Teacher curricula for intimate examinations.

## Introduction

Physicians are required to have fundamental skills in performing pelvic examination, a common gynaecological procedure [1,2]. Therefore, medical students need to develop proficiency in performing these examinations. Obstetrics and Gynaecology (OBGYN) residents play a vital role in medical students’ education, serving as key information and educational sources [3]. Notably, students often perform more pelvic examinations with residents than with senior doctors, highlighting the importance of residents’ involvement in students’ clinical training [4]. Internationally, several countries have recently updated policy and guidelines for intimate examinations, such as the U.S. Department of Health and Human Services [5], and the UK’s General Medical Council [6], redefining this teaching by mandating transparency, explicit written consent, and appropriate supervision. These developments underscore the importance of residents facilitating safe and ethical clinical teaching.

Teaching physical examinations to students can be seen as a straightforward task [7]. However, pelvic examination teaching extends beyond technical and procedural aspects [2], requiring more communication, proficiency, and sensitive doctor-patient interactions, as it is an intimate examination [8]. Intimate examinations include examination of genitalia, breasts, and rectum [7]. Pelvic examination includes inspecting the vulva, performing a speculum examination, cervix inspection, and a bimanual examination of the pelvic organs [9]. Bimanual refers to two fingers in the vagina and a hand on the abdomen to assess pelvic structures by touch. Pelvic examination involves objective (speculum examination) and subjective (bimanual examination) components, alongside patient communication for patient comfort.

Various patient, student, and physician factors and their complex interactions influence pelvic examination performance in clinical environments [1]. Additionally, the OBGYN clinical setting poses unique challenges to residents in their teaching roles [10]. Important patient factors relate to the fact that pelvic examinations can be traumatic and painful if performed without adequately addressing women’s fears or sensitivities [11]. Despite its recognized need, women often find pelvic examination embarrassing [1], and some express reluctance to be examined by medical students [12,13]. Although most women accept medical students’ participation out of altruism for contributing to future physician training [14,15,16], refusal commonly stems from confidentiality concerns [16] and privacy issues [15].

Student factors include uneasiness and concerns over causing patient discomfort, leading them to perform hurried examinations [13,17]. They may struggle for the right words while ensuring patient safety and comfort [2]. Finally, resident factors include residents lacking formal training or established frameworks to guide medical students in pelvic examinations [18] and limited teaching experience in this [8,19]. Furthermore, patients’ medical care takes precedence over their teacher role [19]. Also, residents may face conflicts balancing their own learning with supervision responsibilities, and when struggling with time constraints and building their own competencies, they are less likely to allow students to perform pelvic examinations [15]. During pelvic examinations, the visual inaccessibility of the structures being examined poses a challenge and potential distress for residents supervising students, especially novices [13,17]. Physicians must balance students’ learning needs with ethical obligations [20] by fostering collaborations and conversations between students, women, and residents, grounded in mutual respect, empathy, and professionalism.

Despite the involvement of multiple factors and their complex interactions, little is known about residents’ experiences in conducting clinical teaching and supervision of intimate examinations [7]. Given residents’ key role in medical student education [21], this gap highlights the need to explore residents’ experiences of supervising pelvic examinations. For this purpose, we drew on the Cognitive Apprenticeship Model (CAM) as a lens [22]. This framework is designed to make experts’ cognitive processes visible in practice, and consists of four key components: content, method, sequencing, and sociology [22]. The method component encompassing six key steps—modelling, coaching, scaffolding, articulation, reflection, and exploration—illustrates how residents can demonstrate professionalism, provide feedback, gradually withdraw support to foster independence, and encourage reasoning and self-reflection. Understanding residents’ supervision of pelvic examinations through this lens can highlight the interplay between patients, students, and residents in authentic clinical settings. In this context, our study investigates the research question: *What are the experiences of OBGYN residents regarding their role in supervising medical students performing pelvic examination on patients?* Situating residents’ perspectives within CAM allows this study to inform faculty development and Resident-as-Teacher curricula, while also providing transferable insights for the supervision of intimate examinations across medical specialties.

## Methods

### Study Design

This qualitative study aimed to thoroughly investigate the residents’ supervision of medical students, seeking a comprehensive understanding of their experiences while guiding students in pelvic examinations [23], using one-on-one in-depth interviews. It strived to comprehend various complexities by providing a rich portrayal of social and human aspects inherent in this supervision [24].

### Research Setting

This qualitative research was conducted within the OBGYN Division at KK Women’s and Children’s Hospital, which is Singapore’s largest public hospital for women and children. OBGYN residents undergo residency training modelled on ACGME-I standards [25]. Residents actively teach and supervise medical students in clinical settings, fulfilling their roles as educators as explicitly highlighted in the ACGME core competencies. Many residency programs worldwide adhere to ACGME-I standards. Hence, OBGYN residents in other countries may share similar experiences to those in Singapore.

### Participants

Residents from the OBGYN Division, who had supervised medical students in pelvic examinations, were recruited using purposive sampling. Diverse characteristics including age, gender, ethnicity, nationality, university attended, years of OBGYN experience before residency, and prior educational exposure to pelvic examinations were considered (Appendix 1). Thematic sufficiency, indicating no new information was identified for theme development, guided the final sample size of ten [26]. This is further explained in the Data Analysis section.

### Data Collection

Data were collected through depth interviews [24] conducted in pre-booked meeting rooms at KK Women’s and Children’s Hospital, ensuring a quiet, distraction-free environment [23]. Informed consent for recording and transcription was obtained before assigning participants unique alphabet codes for anonymity. Semi-structured interviews conducted in English were guided by a pre-developed CAM-based interview guide (Appendix 2), using open-ended questions [27]. Interviews were audio recorded. A prior pilot interview was conducted. Flexibility was maintained to explore emerging issues [24]. Researchers maintained field notes on participants’ impressions and non-verbal cues [28].

### Data Analysis

CSP transcribed the interviews verbatim, immediately after each interview [27]. Participant identifiers were omitted from the transcripts. Interviews and analysis were conducted concurrently. This iterative process of comparing new codes with existing ones identified new concepts. By the eighth interview, no additional information for concept and theme development was identified. Another two interviews were conducted to confirm these patterns, reinforce existing themes, and ensure rich data. CSP performed a six-step thematic analysis [29] to code the data and develop themes. The steps, including coding and theme development, were discussed with JF and PK in regular meetings. The steps included: 1) familiarization by ‘repeatedly reading’ data to recognize meanings and patterns. 2) Generating initial codes by noting basic information from raw data and assigning codes that likely represented their meaning. Generated codes were categorized based on similarity, and supporting quotes were highlighted. Codes were modified or new codes added as the study progressed. 3) Searching for themes by organizing the code groups to formulate themes. Initial coding was conducted inductively, allowing themes to emerge from residents’ narratives. CAM served as a sensitizing framework for interpreting emerging themes using its four components. 4) Reviewing themes included constructing a thematic map by reading all extracts for each theme to ensure coherence. 5) Defining and naming themes by discerning the ‘essence’ of each theme, capturing its aspect of the data, and 6) Producing the report, including sufficient extracts to construct a rationale for the research question [29].

### Ethical Consideration

Ethical approval was granted from SingHealth Centralised Institutional Review Board (SCIRB) (CIRB reference number: 2023/2490). All participants signed written informed consent. Participation was voluntary, and withdrawal was permitted without explanation and consequence. The final report anonymized all data, ensuring confidentiality (Creswell & Creswell, 2023).

### Reflexivity

The principal investigator (CSP) is familiar with the resident’s role and challenges in medical education. To minimize power differentials, interviews were conducted by the co-principal investigator (JJLI), a Family Medicine Consultant with no direct ties to the residents. This allowed open sharing of experiences and challenges, while rigorously maintaining confidentiality. Residents were reassured that participation would not affect evaluations. CSP’s own experiences in supervising medical students could potentially affect the interpretation of residents’ experiences. Maintaining an audit trail helped reflect on the study process and the role and influence of individual researchers [30]. Other members of the research team reflected a mix of both insiders and outsiders to the research setting. JF, as an outsider to both the research setting and the phenomenon under investigation, enabled critical questions to be introduced during team reflexivity meetings. For example, during theme development, CSP interpreted student struggles with the technical aspects of speculum examination as a skills deficit, whereas JF helped see the influence of communication and confidence. This iterative reflexive process shaped the themes and enhanced analytical rigor.

## Results

The findings are organized into two thematic categories: 1) Navigating Pelvic Examination Nuances with Residents’ Supportive Strategies, and 2) Dearth of Opportunities for Students to Perform Pelvic Examination.

### Thematic Category 1: Navigating Pelvic Examination Nuances with Residents’ Supportive Strategies

This thematic category highlights the specific challenges students faced when performing pelvic examinations and the detailed strategies residents used to guide them.

#### Structural Nuances in Pelvic Examination

This theme connects students’ struggles, patients’ discomfort, and residents’ interventions. Residents noted that students often struggle with the technical aspects of pelvic examinations, lacking proficiency with speculum handling, which can cause patient discomfort and pain. As one resident commented, *“they [students] do not know how to handle the speculum… inserting it halfway and opening it before reaching the end … causing patients discomfort… after they are done … they do not close it before pulling out… pinch the patient’s skin… causing pain.” (P7)*

To prevent patient discomfort or complaints, residents frequently intervened, stating that “student’s *success… to find a cervix… is not high… [residents] cannot let them try… very long… cannot see what’s going on… after some time… me [residents] taking over… less beneficial for the student…” (P6). In contrast, residents noted that the bimanual exam (BME) is “more straightforward… cannot fail.”* (P6)

However, as bimanual examination is a “blind” procedure, residents struggled to understand what the students were palpating. This uncertainty often led residents to either repeat the examination themselves to confirm findings or restrict student participation entirely, with one resident stating, *“I prefer to do it myself.”* (P5)

#### Resident Strategies for Navigating Pelvic Examination Nuances

In response to these challenges, residents developed supportive strategies, including briefing, coaching, and debriefing. Before a patient encounter, residents walked students through the steps of pelvic examination, familiarizing them with the speculum. During the examination, they provided verbal, step-by-step guidance.

Residents, as students’ near-peers, were able to provide constructive feedback during debrief, tailored to students’ level. One resident described, *“when the patient is changing… [residents] let the student know what went well, how they [students] could have adjusted, what could have been done better.” (P4)* Another resident offered a valuable tip: *“if you can’t find a cervix… remove your speculum, do a vaginal examination, use fingers to feel the cervix and then go again…” (P3)*. One resident, drawing from personal experience, provided clues for increasing the chance of cervix visualization, stating, “if patient has a previous caesarean section, angle the speculum upward” *(P4)*. Another resident recommended, “use a smaller speculum if you see an anxious patient” *(P3)*.

Overall, residents aimed to optimize their supervisory role by actively supporting students through the complex aspects of pelvic examinations and guiding them toward independence using some CAM components. However, this was often hindered by limited opportunities for students to practice.

### Thematic Category 2: Dearth of Opportunities for Students to Perform Pelvic Examination

This thematic category explores factors limiting students’ opportunities to practice pelvic examinations and residents’ efforts to mitigate them. Gender dynamics played a role.

#### Creating Pelvic Examination Opportunities

Residents noted that male students had fewer opportunities to observe and perform pelvic examinations, as more patients declined their participation. This was particularly true for *“younger patients… more conservative ones with husbands here, or with religious or racial beliefs, [patients] don’t want even male doctors, not just male medical students” (P2)*. To increase the chances of consent, a female resident paired male students with male residents, noting *that “female doctor with a male student, they [patients] are likely to say no” (P6)*. Overall, residents were willing to support students exhibiting keen interest, stating, *“If you [students] are keen, I will help you…, we will find a way to let you try your hands on speculum examination” (P5)*. Residents frequently observed that students were hesitant and uncertain, attributing this to the limitations of the students’ training with “lifeless” pelvic models. One resident explained, *“lot of times they [students] are hesitant, apprehensive… [students] check five times whether or not the patient has pain… and this behaviour hinders, and the patient is like, just let the doctor do it” (P10)*. Residents felt less confident involving unprepared students, with one stating, *“[students] don’t know how to hold a speculum, and they’re keen to proceed with an examination…” (P2)*.

One resident commented that effective communication could improve the chances of obtaining patient consent, a major hurdle. The consent rate improved when residents changed their phrasing to, *“can we… examine you together,” instead of, “can my student examine you?” (P6)* Additionally, clinical indications for pelvic examinations being scarce, the opportunities were limited, as *“not every patient requires an examination… it’s quite opportunistic whatever the student gets to do.” (P4)*

#### Alternative Solutions and Systemic Barriers

This theme describes residents’ alternative efforts and ideas. To address the lack of opportunities within systemic limitations, some residents taught students on anesthetized patients undergoing gynaecological surgery. This setting, with explicit consent, enabled structured coaching without patient discomfort. One resident described how *“under GA… they [students] see me going through the steps… in the clinic, I would get students to do the speculum first… a little bit different for the two different settings” (P1)*. This approach was limited by the lack of real-time patient feedback.

Residents identified systemic challenges, including time constraints and overcrowded clinics, as significant barriers to their supervisory role. *“The biggest constraint is time … so many patients in the clinic. It is hard to bring them [students] through things when you’re fighting against time” (P8)*. To improve this, residents suggested implementing protected teaching time, standardized protocols, and formal “train-the-trainer” sessions to enhance their teaching and supervision skills. They also proposed exploring alternative teaching modalities with their limitations. One resident said *“Artificial Intelligence … is a great alternative, …. but lacks tactile component’(P1)*. This resident, trained in the United Kingdom, commented “female volunteers allowed medical students to perform these examinations on them … in a conservative Asian context … it is more difficult” *(P1)*.

Some residents commented that awareness about the students’ curricula and learning objectives would boost their supervisory roles, stating *“If we [residents] knew their requirements… at the end of the posting, [students] should at least know how to do a vaginal exam… do speculum… if we [residents] knew that they [*students’*] must do such things, then we [residents] will be on the lookout…” (P10)*.

## Discussion

Despite challenges posed by the intimate and sensitive nature of pelvic examinations, residents in this study employed strategies consistent with the Cognitive Apprenticeship Model (CAM), particularly the six steps of the “methods” component [22], facilitating effective supervision. These strategies included, most critically, modelling, coaching, scaffolding, reflecting, and exploring [22], each contributing to students’ growing independence in performing pelvic examinations. Residents modelled professionalism during patient contact, provided coaching and scaffolding tailored to student proficiency and task complexity, encouraged articulation, and reflected on ways to explore effective pelvic examination teaching. Sociology addressed consent, ethics, and cultural norms involved in these examinations, as well as system challenges such as time constraints, and busy clinics with long patient waiting times. A myriad of factors posed hurdles to residents’ supervisory experiences in this study, some of which were difficult to change. Beyond the OBGYN context, the insights from this study are likely transferable to other medical specialities (breast, rectal, and urogenital), which teach and supervise intimate examinations, with attention to ethics, patient comfort, and communication. CAM may offer a useful structuring lens for guiding supervision of intimate examinations across these clinical specialities by making reasoning and professional behaviours visible, within the limitations of the context.

Limited literature exists on residents’ training in pelvic examinations, despite ACGME’s emphasis on structured programs incorporating standardized skills assessment, as highlighted by Cullimore et al [21], thereby aiding the design and development of structured, modular, and context-sensitive Resident-as-Teacher (RaT) programs [31]. While RaT frameworks offer broad guidance for their curricula, Cognitive Apprenticeship provides a granular lens, making explicit how supervisory strategies are calibrated to task-specific characteristics. Residents calibrated speculum and bimanual examination teaching based on visibility and technical complexity, as well as patient factors, and clinical constraints. Our findings suggest that explicit use of CAM, especially the method components, can be actionable in the design of robust RaT training to aid residents’ supervisory roles across specialities teaching intimate examinations. Integrating these into faculty development can help residents navigate complex clinical environments and encourage them to take on future faculty roles.

Residents in this study identified content gaps with students lacking technical skills and struggling with speculum examination, thereby undermining its primary objective, namely cervical visualization. Residents provided scaffolding by aligning support with students’ existing knowledge [22]. As students’ near-peers, residents exemplified scaffolding, to prevent pitfalls through practical, experience-based tips, reflecting near-peer teaching. Difficulties like improper speculum handling, insertion angle, and timing of opening/closing were overcome by residents’ stepwise coaching to improve cervix visualization. RaT programs could highlight such near-peer teaching opportunities, using CAM components. Furthermore, building on our findings, future research could explore how observable behaviours, feedback targets, and levels of supervision for each step could be set as an input for OSCE checklists and EPAs. Conversely, related to CAMs’ sociology component, residents in this study experienced a dearth of opportunities to supervise students performing pelvic examinations, because of gender and consent issues, insufficient indications, and residents’ intervening in difficult situations. Effective supervision of bimanual examination using CAM components was limited, as residents intervened when students were slow, uncertain in palpation, or to reduce patient discomfort, resonating van den Einden et al. [15].

Additionally, gender bias limited pelvic examination opportunities for male students, and hence supervisory opportunities for residents. Male students had fewer chances than female peers, aligning with studies showing higher patient refusal rates for male students [32,33,34,35]. Cultural, ethnic, and religious backgrounds, and patient age, contributed to this phenomenon. Unlike Akkad et al. [36], who found tutors less supportive of male students seeking consent for intimate examinations, residents in this study were inclined to apply the “exploratory” component of CAM to students of either gender, demonstrating genuine enthusiasm and a desire to teach pelvic examination.

Unfortunately, our residents noted overall consent issues. This aligns with previous research that found patients were more concerned about training levels, clinicians’ reputation, communication skills, and technical ability than gender [37,38]. This explains why patients declined medical students, which residents mitigated by introducing them as “future colleagues.” However, van den Einden et al. [15] cautioned against terms like “future colleague”, as blurring the student’s identity raised an ethical dilemma. Liu et al. [39] stressed explicit student introductions and role clarification in patient care.

Residents reported student challenges including unpreparedness, uncertainty, hesitancy, and low confidence in performing pelvic examinations, which may cause patients’ uneasiness, and therefore declined examinations [40]. From CAM’s sociology perspective, residents encouraged reflection on attitudes, confidence, and patients’ reactions, alongside requesting standardized training protocols for students’ pelvic examination preparation, addressing CAM-content (knowledge/skills) [22]. Low confidence was attributed to limited pelvic examination opportunities and training using simulated pelvic trainers, that helped facilitate the examination process but lacked authentic human interaction [41]. A resident shared an overseas teaching perspective involving gynaecological teaching associates or female volunteers, who allow medical students to perform pelvic examination on them and provide real-time human feedback, improving student competence, communication skills [8], and interactive support [42], while enabling a comfortable and safe learning space [40], and cross-assessment of competence for other intimate examinations [8]. Thus, residents employed reflection and exploration to discuss this potential locally, within sensitive cultural constraints.

To address limited opportunities, our residents assisted medical students in pelvic examinations on patients under general anaesthesia with consent. Through relaxed muscle state, this approach enabled better assessment of pelvic anatomy and clinical findings, without patient discomfort, which may be challenging when patients are awake [39]. It allows a slower instructional coaching approach, with residents modelling exact steps, followed by students performing pelvic examinations themselves. Residents encouraged students to articulate findings and provided feedback, thus employing different CAM components. With UK’s General Medical Council 2024 guidance, necessitating explicit patient consent for all aspects of intimate examinations, including examination under anaesthesia, opportunities may be further constrained [6]. Additionally, residents considered teaching associates or virtual reality models, “exploring” step-by-step modelling and coaching to perform speculum and bimanual examination. Real-time feedback from these models supports progression towards self-directed and independent practice (scaffolding). However, regardless of simulation innovations, residents still need to calibrate supervision within authentic clinical contexts (CAM’s sociology component), judging clinical appropriateness, navigating consent, and responding to patient cues and student anxiety.

With evolving international guidance governing intimate examinations this is crucial. Recent updates from the US DHHS for Medicare & Medicaid Services [5], and UK’s GMC [6] on intimate examinations and chaperones, emphasize explicit consent, transparency, and appropriate supervision when learners participate in these examinations. These developments reflect a broader shift towards safer and more ethically structured training environments. Our findings suggest that residents already play a central role in operationalizing these expectations within everyday clinical teaching, balancing comfort, student learning needs, and clinical pressures.

In this study, only residents’ perspectives were explored. Including patients’ narratives in future research could complement residents’ accounts by incorporating experiences of those directly involved and further optimize intimate examination teaching practices. Medical students’ and senior physicians’ views could also be explored. Furthermore, purposive sampling may have attracted highly motivated participants, and the study included only one setting. Data from other settings involving diverse groups of participants may yield additional insights. Additionally, the Cognitive Apprenticeship Model, with its cognitive focus, provided a perspective into specific aspects of resident supervision, but might have overlooked elements highlighted by other frameworks. Exploring frameworks other than CAM could provide perspectives into specific aspects of supervision. The researchers nonetheless upheld openness during the interviews and analysis of diverse experiences, including participants’ feelings and emotions, applying CAM as a sensitizing rather than a rigid analytical framework.

## Conclusion

This study highlights OBGYN residents’ challenges in supervising medical students performing pelvic examinations. CAM components can be explicitly mapped to teaching pelvic examinations, and calibrated to student competency and task complexity. These findings have practical implications for Resident-as-Teacher programs and faculty development, providing clear guidelines, expectations and structured training for intimate examination teaching and supervision.

## Practice Points

- Residents supervising intimate examinations face distinct challenges due to its sensitive and nuanced nature: yet, structured frameworks to guide supervision can help guide Resident-as-Teacher (RaT) programs.
- Lack of specific RaT training for OBGYN residents can create tension between patient comfort, ethical considerations, and effective supervision.
- Applying the Cognitive Apprenticeship Model as a lens can enhance residents’ supervision and teaching of pelvic and other intimate examinations beyond OBGYN.
- Exploring protected teaching time, formal support, and innovative tools such as AI or VR might strengthen resident supervision and student learning outcomes for intimate examinations.

## Appendices

**Appendix 1:** Participant characteristics.

DEMOGRAPHICS		

Total Participants	n	10

Sex	Male	3

	Female	7

Age	(25–30 years)	5

	(31–35 years)	5

Ethnic group	Chinese	7

	Indian	1

	Malay	1

	Eurasian	1

Previous Study	Local University	8

	International Universities	2

Years of Practice before joining OBGYN residency	1	5

	2	4

	3	1

Number of residents with prior educational exposure to pelvic examination	Gynaecological teaching associate	1

	Simulation in pelvic examination	0

	Pelvic examination workshop with mannequin	10

	Artificial Intelligence/Virtual Reality	0

**Appendix 2:** Interview Guide.

**Notes for the Interviewer**

Interviewer Preparations Prior to the Conduct of the Interview

1. Please familiarize yourself with the research aims by reading the research proposal. 2. Be familiar with the general interview approach.

**Advice before Starting the Interview**

1. Introduce yourself. 2. Explain in broad terms the purpose and goals of the study. 3. Ask the participants if they have any questions. 4. Check and confirm the consent approval with the participants. 5. Inform the participants that this interview will be recorded. 6. Open the interview with questions that the participants can answer confidently. 7. Please refer to the suggested opening questions.

**Open-Ended Semi-structure Interview Questions**

**Please state your residency training year**

How often do you supervise medical students in your clinic and how often do you supervise them performing Pelvic Examination (PLE) on patients? Can you describe or share some thoughts about your role as a resident doctor supervising medical students performing PLE on patients? (What are some positive or negative experiences you experienced?) Can you guide me through a “typical” supervision of a PLE: e.g., what do you do, which steps do you take, and what are your considerations for supervising such examination and what are some feelings involved? What are some of the key challenges in providing supervision for “medical students performing a PLE on patients”? What are your thoughts for improving the training or support for effective supervision of medical students performing PLE on patients?

**Probing Questions**

**Modelling and Coaching**

Can you tell us more on the coaching strategies that you may be using while supervising “medical students performing a PLE on patients”? Please describe how you provide feedback while supervising “medical students performing a PLE on patients”?

**Scaffolding and Sequencing**

Can you comment on how you guide the medical students through different aspects of performing a PLE on patients including introducing them to more complex steps?

**Articulation**

How do you get the medical students to describe the feelings or emotions they experience while performing a PLE on patients?

**Reflection and Exploration**

What happens after the PLE is performed? How do you feel after the supervision is done? What are your thoughts? With these reflections how do you to prepare for the next session?

**End of Interview**

1. Thank the participants for their cooperation and participation in the interviews. 2. Assure the participants of the confidentiality of their responses. 3. Let the participants know how to get in touch with the researchers if they need to raise any issues or concerns later. 4. Let the participants know that a summary of their comments will be sent to them at later date for clarifications or additional comments.
